# Traumatic Macular Hole: Diagnosis, Natural History, and Management

**DOI:** 10.1155/2019/5837832

**Published:** 2019-03-19

**Authors:** Greg Budoff, Neelakshi Bhagat, Marco A. Zarbin

**Affiliations:** Rutgers-New Jersey Medical School, Institute of Ophthalmology and Visual Science, Rutgers University, New Brunswick, NJ, USA

## Abstract

Traumatic macular hole occurs most often in young men and can present after various types of injuries. Traumatic macular holes result from anteroposterior and tangential vitreoretinal traction and may exhibit concurrent additional pathologies such as Berlin's edema and subretinal fluid. Optical coherence tomography can play an essential role in patient management both at presentation and during follow-up. Initial management consists of observation, but macular hole repair can be performed if spontaneous resolution does not occur. Upon macular hole closure, vision may improve, on average, by two lines or more but may be limited by associated macular pathology.

## 1. Introduction

A macular hole (MH), a full-thickness defect of the neurosensory retina at the fovea, occasionally can result after trauma. The first case of traumatic MH was described by Knapp in 1869 [1]. While idiopathic MHs occur more commonly among women older than 65 years of age [2], traumatic MHs are found more often in young men in their early twenties [3, 4]. Because traumatic MHs frequently are associated with sports and work-related accidents, they occur predominantly in younger persons. The incidence of traumatic MHs is 1.4% in closed-globe trauma [5] and 0.15% in open-globe injuries [6]. Traumatic MHs also can occur following laser injury [7], surgical trauma [8], lightning strikes [9], and shock with electrical current [10].

Vision at presentation ranges from Snellen 20/30 to 20/400 [3, 4]. The functional prognosis often is uncertain due to the accompanying trauma-induced retinal pathologies, such as commotio retinae, vitreous hemorrhage, retinal hemorrhage, choroidal rupture, retinal pigment epithelium (RPE) damage, and subretinal choroidal neovascularization and fibrosis.

Treatment includes observation, as spontaneous closure has been reported [11–15], as well as surgical closure with vitrectomy if necessary.

## 2. Pathogenesis

In contrast to idiopathic MHs which develop gradually over months [16], MHs more commonly form immediately after trauma [17] although there are reports of holes developing weeks following injury [14]. Two hypotheses exist to explain the development of traumatic MHs, and these are similar to their idiopathic counterparts, in that tangential and anteroposterior vitreoretinal traction are the main culprits.

One theory proposes anteroposterior vitreous traction on the fovea causing true loss of foveal tissue. This scenario could be thought of as a trauma-induced force vector with a component orthogonal to the corneal surface resulting in anteroposterior compression of the globe with equatorial globe expansion followed by a rebound contrecoup resulting in vitreofoveal traction. However, a flaw with this theory is that many studies have not found a posterior vitreous detachment (PVD) in traumatic MHs nor has evidence of vitreomacular traction consistently been observed with optical coherence tomography (OCT) [4, 6, 17]. Additionally, following surgical closure vision typically improves at least two lines [6, 17], which would not likely be the case if true loss of foveal tissue had occurred. However, Yamashita et al. reported a hole developing a few days following trauma which showed OCT evidence of vitreomacular traction. The hole closed spontaneously one month later after the vitreous fully detached from the fovea [14]. Thus, it is possible that anteroposterior vitreous traction without true foveal loss can be causative and may contribute to MH formation in some cases.

Likely, a more frequent cause proposed by Johnson et al. [17] is as follows. Blunt trauma to the eye occurs in the axial direction, causing a decrease in the axial length of the globe. Subsequently, there is an expansion of the globe along its equatorial dimension. This expansion results in tangential vitreoretinal forces that are transmitted to the macula, with centripetal forces separating the neurosensory retinal layers at the fovea, resulting in a central defect without any loss of foveal tissue.

It is likely that a combination of anteroposterior vitreoretinal and tangential retinal traction is the cause in many cases. However, given that commotio retina sometimes resolves with substantial photoreceptor atrophy and given that the predominant cell type in the foveal center is the specialized cone photoreceptor, loss of foveal photoreceptors may cause full-thickness macular holes in some patients after globe trauma [18]. In these cases, closure of the hole may not be possible, and, even if it is, recovery of precision vision is not likely to occur.

## 3. Examination

**Figure 1:**
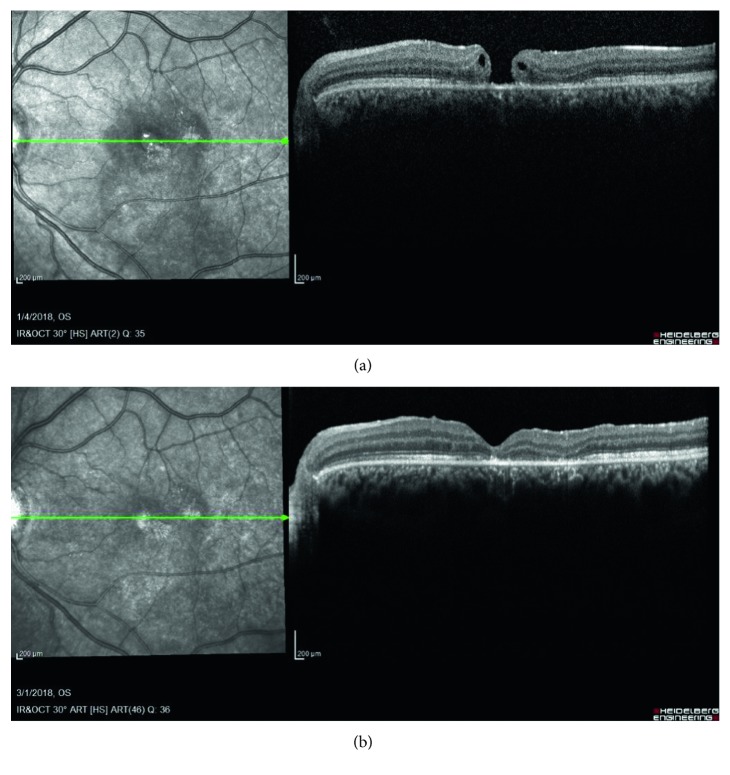
An 18-year-old male, punched in the face, presented with visual acuity of 20/60 with Berlin's edema and a full-thickness macular hole. He underwent surgical repair of the hole with pars plana vitrectomy, induction of a posterior vitreous detachment, and internal membrane peel, two months later. (a) OCT, two months after presentation, shows a full-thickness macular hole with intraretinal fluid. (b) OCT, two months after surgical repair, shows a closed macular hole and parafoveal disruption of the ellipsoid zone. Visual acuity was 20/40.

**Figure 2:**
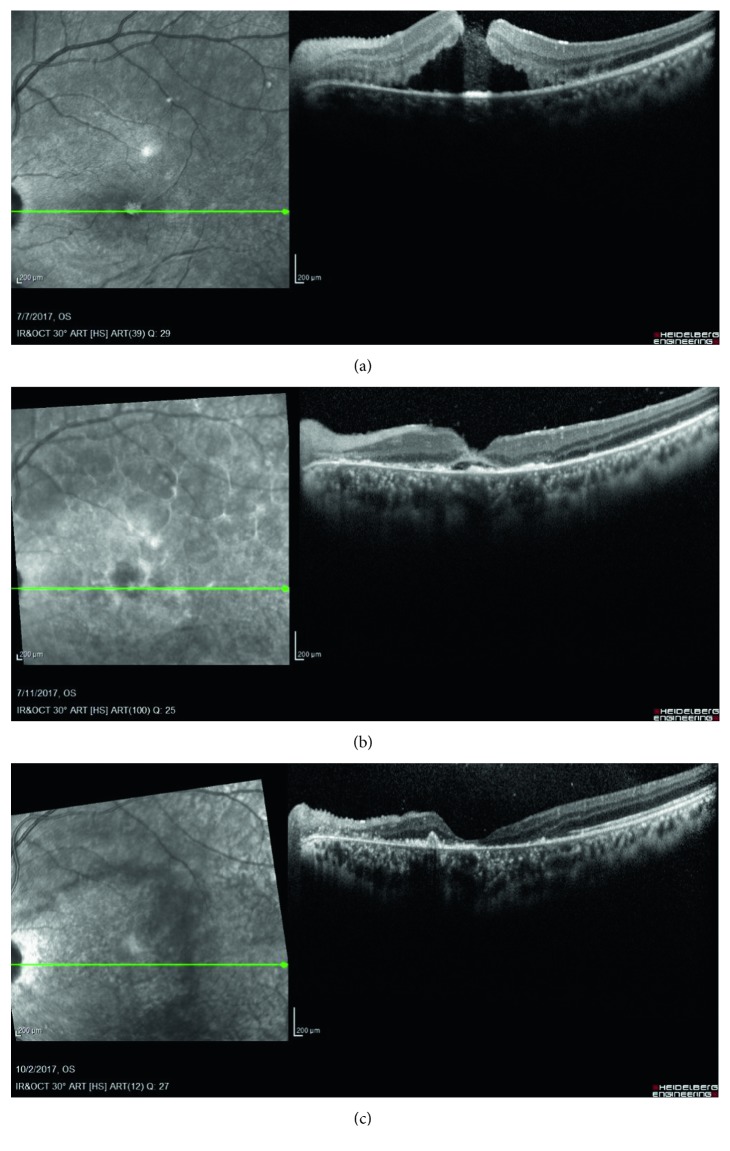
A 19-year-old male who sustained a firework explosion to his face presented with a macular hole that resolved spontaneously within three months of follow-up. (a) OCT at presentation shows a full-thickness macular hole and extensive subretinal fluid. Visual acuity was 20/400. (b) OCT a week after presentation shows a closed macular hole with some residual subretinal fluid. Visual acuity was 20/400. (c) OCT at the three-month follow-up visit shows a closed hole with resolved subretinal fluid but with extensive outer retinal atrophy and an increase in subretinal deposits. Visual acuity was finger counting.

Patients usually complain of decreased vision and metamorphopsia. The vision may range from 20/30 to 20/400. If there are no additional injuries, there is no afferent pupillary defect (APD). On clinical examination, a round or ellipsoid full-thickness defect of the neurosensory retina in the macula is seen with a rim of subretinal fluid (Figures 1 and 2). Retinal edema and subretinal hemorrhage may be evident in many eyes if examined immediately after the traumatic event. Posterior vitreous detachment is absent in most cases. Usually, the traumatic MHs involve the foveal center, but they can have an eccentric location, particularly if they arise from surgical trauma [8].

The Watzke–Allen sign is usually positive [9]. A laser aiming beam directed in the central defect may not elicit any perception of the light spot. The Amsler grid usually will show central metamorphopsia [9]. A fluorescein angiography may reveal late central hypofluorescence or hyperfluorescence [19], depending on the RPE changes that have developed. OCT shows a full-thickness defect in the neurosensory retina occasionally with a cuff of subretinal fluid, usually without PVD or evidence of vitreomacular traction [12, 15]. Intraretinal cysts may be noted in some cases, particularly at the edge of the full-thickness retinal defect [15].

Fundus autofluorescence (AF) has been used to stage and monitor idiopathic MHs [20–22]. Relative hypoautofluorescence is normally noted at the fovea due to the presence of lutein and zeaxanthin pigments that attenuate the RPE autofluorescence [21, 22]; in eyes with MH, however, the lack of these pigments results in hyperautofluorescence which disappears with successful anatomic closure. Stellate hyperautofluorescence with striae may be associated with cysts in the outer plexiform layer and present with better VA and lower stage MH than eyes without a stellate appearance [20].

A large study comparing OCT findings in traumatic MHs to idiopathic MHs was completed by Huang et al. [4]. They found that traumatic MHs are more eccentric, or less circular, than idiopathic MHs, with the horizontal diameter significantly longer than the vertical diameter. This eccentricity is consistent with the tangential traction theory of development. As the equatorial horizontal diameter of the globe itself is longer than its vertical diameter [23], a stronger shearing force is likely applied at the macula in the horizontal meridian, resulting in the greater horizontal hole diameter observed in traumatic MHs. Huang et al. also reported significant differences in the average size of traumatic versus idiopathic MHs—a larger basal diameter and basal area of 1338.45 *μ*m and 176.85 *μ*m^2^ versus 958.57 *μ*m and 77.92 *μ*m^2^, respectively, with retinal thickness much lower at 248.4 *μ*m compared to 408.8 *μ*m at the edge of the defect. They noted that there was no correlation between visual acuity (VA) and size of traumatic MH. This finding is in contrast to idiopathic MHs, the diameter of which is positively correlated with worse VA. No evidence of PVD or operculae was seen on OCT in this study.

Teng et al. using OCT angiography showed an association between decreased subfoveal choroidal blood flow in eyes with idiopathic MHs compared to healthy fellow eyes [24] but, to date, this has not been studied in eyes with traumatic MHs.

## 4. Management

### 4.1. Observation

Most spontaneous closures of traumatic MHs occur in children or young adults [12, 14, 15]. Miller et al. described a 50% spontaneous closure rate in children and a 28.6% rate in adults in a long-term follow-up series of traumatic MHs [12]. The holes closed spontaneously at a median of 5.6 weeks, and none closed without intervention after 67.3 weeks [12]. Other series also report a spontaneous closure rate of 37%–44% within two months after injury [14, 15]. OCT plays an important role in the management of traumatic MHs to delineate the anatomic details of the defect, associated retinal pathology, and also to assess its progression objectively on a weekly or monthly basis.

Many investigators have noted that smaller traumatic MHs have a higher chance of spontaneous resolution [12, 14, 15]. The majority of such eyes have no PVD [14, 25]; the posterior hyaloid may play a tamponading role to prevent fluid from entering the hole. Chen et al. found that holes with spontaneous closure were less likely to have intraretinal cysts compared to the holes that did not close spontaneously (10% versus 76.5%, respectively) [15]. They suggested that the absence of intraretinal cysts on OCT may predict spontaneous traumatic MH closure. Of note, case reports describe topical nonsteroidal anti-inflammatory drugs, resulting in closure of traumatic holes associated with cystoid macular edema [26].

One proposed mechanism for spontaneous hole closure is glial cell proliferation from the level of the RPE to the basal surface of the hole, thus promoting closure [13]. If there is an element of anteroposterior vitreomacular traction, formation of a complete PVD may also foster closure of the hole. As Berlin's edema resolves, the edges of the hole may return closer to the RPE, resolving the central full-thickness defect.

There is no consensus on when to operate on a traumatic MH that does not close spontaneously. As noted above, many reports describe spontaneous closure within two months of trauma [12, 14, 15]. Additionally, Miller et al. noted that traumatic MHs that underwent delayed vitrectomy, on average approximately one year after presentation, were less likely to close than those operated on earlier [12]. Thus, the vitreoretinal surgeon should strive to balance waiting long enough for spontaneous closure but not so long that the likelihood of an anatomically successful outcome is materially reduced. Waiting one to three months before intervening with surgery seems prudent.

### 4.2. Surgery

#### 4.2.1. Basic Technique

Vitrectomy to surgically induce the closure of idiopathic MHs was described originally by Wendel et al. as comprising five steps: (1) pars plana vitrectomy; (2) induction of a posterior vitreous detachment (PVD), if not present; (3) epiretinal membrane peel; (4) fluid-gas exchange; and (5) one week of occiput-up positioning [27]. They reported a 73% overall single-operation anatomic success rate; patients with symptom duration less than six months had an 80% overall single-operation anatomic success rate. Since the introduction of this technique, many variations have been described in an attempt to improve the anatomic success rate of traumatic MH closure.

Surgical adjuvants such as TGF-beta 2, platelet concentrate, and serum were used to close idiopathic MHs following Kelly and Wendel's report [28]. Subsequently, these adjuvants were used in the repair of traumatic MHs. The adjuvants are placed into the hole following the fluid-air exchange and prior to the instillation of the tamponade agent. One proposed mechanism for the use of these adjuvants is that they could assist in the formation of a chorioretinal adhesion that would act as a barrier preventing more fluid from entering the subretinal space [29], thus enabling MH closure.

#### 4.2.2. TGF-Beta 2

Rubin et al. utilized TGF-beta 2 in a series of 12 eyes with a traumatic MH and noted a 67% (8/12) single-operation success rate, which improved to 92% (11/12) after repeat surgery in the four eyes that failed initially [30]. In contrast to the procedure described by Kelly and Wendel, their method called for 24 hours of supine positioning to allow for the TGF-beta 2 to maintain contact with the macula before beginning two weeks of face-down positioning; 67% of eyes gained two lines of vision or more.

#### 4.2.3. Serum

Johnson et al. reported 25 eyes that underwent vitrectomy for traumatic MH, 12 of which received serum as a surgical adjuvant in addition to perfluoropropane (C3F8) tamponade [17]. They noted MH closure in all cases treated with serum, with two or more line visual improvement in 75% of eyes. However, there were no significant differences between the rate of closure or visual improvement between eyes treated with or without serum.

#### 4.2.4. Platelet Concentrate

García-Arumí et al. infused the platelet concentrate over the traumatic MH before infusing sulfur hexafluoride gas (SF6) in a series of 14 eyes [19]. These patients were kept supine for 1 hour after surgery before adopting strict face-down positioning. They reported an 86% (12/14) single-surgery success rate, which improved to 93% (13/14) after the second surgery. Visual acuity improved at least two lines in all eyes in which the MH closed, and at least 50% of eyes had a final visual acuity better than or equal to 20/30.

#### 4.2.5. Internal Limiting Membrane (ILM) Peeling

Kuhn et al. were among the first to report peeling the ILM before the fluid-air exchange in eyes with traumatic MHs in a series of 17 eyes utilizing SF6 tamponade [6]. The single-surgery success was 100% with 94% of patients improving at least two lines of vision and a mean improvement of six lines. Bor'i et al. published a series of 26 eyes that underwent vitrectomy and ILM peeling and reported a 92% single-surgery success rate [31].

#### 4.2.6. ILM Flap Techniques

An inverted ILM flap, initially proposed by Michalewska et al. for the treatment of idiopathic MHs [32], involves an ILM peel circularly some distance away from the hole while keeping the ILM attached to the border of the hole and then directing the inner layer of the ILM flap over the surface of the MH. Abou Shousha utilized this technique for traumatic MHs in a series of 12 eyes with SF6 tamponade [33]. The closure rate was 100% with a median final visual acuity of 20/100. An alternative technique proposed by Morizane et al. [34] has been described for refractory idiopathic MHs. In this method, the surgeon creates a free ILM flap the same size as the hole; the flap is then placed into the MH and covered with a viscoelastic material. The proposed mechanism for both techniques is that the ILM flap contains residual Mueller cells that can use the flap as a scaffold on which to proliferate and subsequently seal the hole [32].

#### 4.2.7. Plasmin-Assisted Vitreolysis

Pediatric patients have very strong adhesion between the posterior hyaloid and the ILM. Plasmin is an enzyme that can hydrolyze fibronectin and laminin, two extracellular matrix proteins responsible for the strong adhesion [35]. Wu et al. injected plasmin intravitreally before vitrectomy with the goal of weakening the hyaloid-ILM adhesion and thus making the surgical induction of PVD easier in a series of 13 pediatric eyes with a traumatic MH [35]. They noted 38% (5/13) of these patients already had a partial or complete PVD at the start of surgery, and in the remaining 62%, inducing the PVD was much easier and required less than 50 mmHg of suction; 92% of patients had their holes close, and 92% achieved two-line or greater visual improvement with 50% achieving a final vision better than or equal to 20/50. No studies have reported using ocriplasmin for traumatic MHs, which was approved for the use of idiopathic MHs 400 *μ*m or less with evidence of vitreomacular traction [36].

#### 4.2.8. Tamponade Agents

SF6, C3F8, and silicone oil all have been used as tamponade agents in traumatic MH surgery with air tamponade also being described for idiopathic MHs [37].

SF6 tamponade has attained as high as 86–100% anatomic success for traumatic MHs with face-down positioning for one or two weeks [6, 19, 33]. Oil tamponade is used uncommonly, and two studies have retrospectively compared silicone oil and C3F8 for traumatic MH surgery [31, 38]. The use of both agents involved two weeks of face-down positioning. Silicone oil has reported anatomic success rates of 67%–90%, while C3F8 has a higher anatomic success rate of 90–92.3%.

Typically, gas tamponade is preferred to silicone oil for the closure of traumatic MHs to obviate the need for an additional surgery to remove the oil, unless face-down positioning would be difficult or impossible as in pediatric cases or incarcerated patients.

### 4.3. Visual Outcome of Traumatic Macular Hole Closure

Whether spontaneous or vitrectomy-induced, traumatic MH closure is generally associated with visual acuity improvement of at least two lines; however, there is potential for greater improvement. Kuhn's series on vitrectomy with ILM peel noted mean improvement of six lines of vision [6].

Trauma can cause photoreceptor and RPE damage, limiting the visual potential of these eyes even with successful MH closure. Many eyes exhibit commotio retinae or faint subretinal hemorrhage immediately after the injury. Rapid resolution of retinal edema and hemorrhage with no clinical sequelae can follow. In many cases of traumatic MHs, however, RPE changes are observed a few weeks after the injury, suggesting RPE and photoreceptor damage. OCT may reveal disruption of the RPE and ellipsoid layers. Johnson et al. alluded to Berlin's edema being associated with photoreceptor and RPE damage and mentioned a few eyes that had significant RPE changes after surgery with final visual outcome ranging from 20/30 to 20/200 [17]. Miller et al. noted no significant difference in final visual acuity in eyes that had foveal Berlin's edema on presentation versus those that did not [12]. Additionally, Miller et al. noted a statistically insignificant trend for better final visual acuity among eyes that had an intact ellipsoid zone on OCT, suggestive of an intact healthy photoreceptor layer, as compared to those that did not [12].

A literature review of traumatic MH studies by Liu and Grzybowski noted an anatomic success rate of 45% to 100% (median, 92.5%) and a functional success (Snellen visual acuity two or more lines improvement) rate of 27% to 100% (median, 84%) [39].

While the vast majority of cases in the literature are secondary to direct ocular trauma with generally good prognoses, two case reports of traumatic MHs secondary to lightning or electrical shock injuries have both resulted in light perception vision and recurrent retinal detachments following pars plana vitrectomy [9, 10].

## 5. Conclusion

Traumatic MH can result from tangential and anteroposterior vitreomacular traction. It may also result from foveal photoreceptor atrophy following commotio retinae. As compared to idiopathic MHs, traumatic holes tend to be more common in younger male patients. Spontaneous closure can occur; however, vitrectomy, induction of PVD, and ILM peeling (with or without an ILM flap to cover the MH) is associated with anatomic success rates of up to 100% [6, 33]. Functional success despite anatomic closure may be limited by trauma-induced concurrent macular pathology.
